# Behavior Change with Fitness Technology in Sedentary Adults: A Review of the Evidence for Increasing Physical Activity

**DOI:** 10.3389/fpubh.2016.00289

**Published:** 2017-01-11

**Authors:** Alycia N. Sullivan, Margie E. Lachman

**Affiliations:** ^1^Psychology Department, Brandeis University, Waltham, MA, USA

**Keywords:** fitness trackers, physical activity, older adults, behavior change, technology, exercise

## Abstract

Physical activity is closely linked with health and well-being; however, many Americans do not engage in regular exercise. Older adults and those with low socioeconomic status are especially at risk for poor health, largely due to their sedentary lifestyles. Fitness technology, including trackers and smartphone applications (apps), has become increasingly popular for measuring and encouraging physical activity in recent years. However, many questions remain regarding the effectiveness of this technology for promoting behavior change. Behavior change techniques such as goal setting, feedback, rewards, and social factors are often included in fitness technology. However, it is not clear which components are most effective and which are actually being used by consumers. We discuss additional strategies not typically included in fitness technology devices or apps that are promising for engaging inactive, vulnerable populations. These include action planning, restructuring negative attitudes, enhancing environmental conditions, and identifying other barriers to regular physical activity. We consider which strategies are most conducive to motivating behavior change among sedentary adults. Overall, fitness technology has the potential to significantly impact public health, research, and policies. We suggest ways in which app developers and behavior change experts can collaborate to develop successful apps. Advances are still needed to help inactive individuals determine how, when, where, and with whom they can increase their physical activity.

Physical activity is broadly beneficial for physical, psychological, and cognitive aspects of health (1–3). Yet, only one in five adults in the US meets the CDC physical activity guidelines of 150 min of aerobic activity and 2 days of muscle strengthening activity per week (4). This trend of inactivity increases with age, as less than half of adults aged 65–74 years and about one-third of adults aged 75 years and older meet these recommendations (5). A sedentary lifestyle is also more prevalent among those with low socioeconomic status (SES) (4). Such vulnerable populations are at increased risk for health problems, and they face unique obstacles to meeting physical activity recommendations (6–9). Making physical activity accessible and feasible for all groups is an important public health objective that is within reach with the right behavioral and environmental supports. Special considerations should be made to address the key barriers in vulnerable, inactive populations such as older adults, especially those low on the SES gradient (10). These include environmental obstacles, such as not knowing where to exercise, time constraints, such as believing there is not enough time to exercise, and social limitations, such as not having support for exercise.

## Incorporating Physical Activity into Daily Life

Walking has gained much interest in recent years as a feasible strategy for increasing physical activity. Walking is a free, low-impact way to meet the CDC recommendation of 150 min of moderate-intensity exercise per week. It can be done just about anywhere, at any time, and does not require equipment or a gym membership. Fitness technology, including fitness trackers and smartphone applications (apps), has recently become publicly available and provides feedback about amount and intensity of activity. Such technology encompasses individual fitness trackers that can stand alone, a fitness tracker that pairs with a companion app, or an app that can be downloaded onto a smartphone without the need for an extra device. The fitness tracker market is currently thriving, with estimates of almost 1.5 billion dollars in revenue last year alone (11). In fact, this market is expected to increase to a five billion dollar industry by 2019 (12). A 2013 analysis revealed that there are over 40,000 health and fitness apps currently available to the public via iTunes (e.g., Map My Walk, Runkeeper, My Fitness Pal) (13), and over half of smartphone users report having downloaded such an app (14).

While fitness technology is already widely used and shows potential for improving public health, there are still many unanswered questions. It is unknown to what extent this technology leads to increases in activity levels over either the short or long term. Further investigation of the usefulness of this technology for sustained behavior change is warranted. To encourage long-term changes in physical activity, the strategies incorporated into technology should include evidence-based techniques derived from behavior change theories. These techniques should directly address the special barriers to increasing activity in vulnerable populations who are at highest risk for a sedentary lifestyle, such as older adults and those low in SES. This includes helping inactive individuals to decide how, where, when, and with whom they can exercise. It is also not clear under what conditions these various devices provide the most accurate data. Some uncertainties remain regarding the best individual trackers, smartphones, or apps, their best locations for placements on the body, and whether accuracy varies across users of different ages and health status (15, 16).

In this review, we examine both the promotion and measurement of physical activity through the use of behavior change strategies and fitness technology. If this technology does facilitate behavior change over the long haul, there is great potential for encouraging population-wide changes in exercise habits and ultimately health. While some studies have used fitness technology primarily to document and measure activity level, others have incorporated it into a behavioral intervention with additional components. We evaluate the presence or absence of specific behavior change techniques derived from cognitive-behavioral perspectives, with a special consideration of which techniques are most useful for sedentary adults. We review recent studies that have examined the extent to which fitness technology, including trackers, smartphones, and apps, provides accurate measurement. Technology that provides valid and reliable data shows a great deal of promise for their application to both research and personal settings. We examine the effectiveness of fitness technology for promoting behavior change and evaluate whether these changes are long lasting. Indeed, recent reports have suggested that activity trackers are used only for short periods of time (17, 18). Some have even suggested that more than half of fitness tracker owners abandon their devices within the first month (18). It is possible that the right behavioral supports would lead to more sustained use, and therefore long-term behavioral changes. Finally, we consider the potential drawbacks and limitations of this technology, as well as their promise for improving public health. This includes a consideration of which populations are less likely to use these monitors, and potential ways to bring this technology to those who could benefit from it the most.

## Strategies for Promoting Behavior Change

Many cognitive factors influence the decision to increase physical activity. Cognitive behavior therapy (CBT) techniques are effective in encouraging increases in exercise throughout the day. This approach can help individuals determine how their internal thoughts guide their exercise behaviors (19). When incorporated successfully into an intervention, CBT techniques can increase physical activity overall, while improving functional health (20). Such strategies that focus on cognitive factors can help inactive people to restructure self-defeating or erroneous thoughts and to develop positive feelings and attitudes toward increasing their activity, and consequently improve their health (19–21). Further, these changes in thinking can lead to increased motivation, making long-term lifestyle changes more feasible (21). Fitness technology often does not include cognitive factors in their programs.

Social cognitive theory (SCT) also provides a useful framework for changing behavior, including exercise (22, 23). This theory suggests that people learn not only from their own experiences but also from watching others. Self-efficacy, or beliefs about one’s abilities to carry out desired behaviors, is a major component of this theory. Perceived self-efficacy is said to be key, as it affects both motivation and actions. Self-efficacy beliefs for exercise are important for lasting changes in physical activity (24, 25). Social factors such as social support or competition are also important within this framework. SCT highlights the importance of expectancies and beliefs for behavior change and suggests that intervening on both beliefs and behaviors is ideal. While fitness technology often focuses on changing behaviors, it typically does not focus on changing beliefs on how to increase activity.

A useful framework for describing behavior change techniques is the Coventry, Aberdeen, and London-Refined (CALO-RE) taxonomy of behavior change techniques (26). This taxonomy provides common terminology for describing health-related behavior change techniques in interventions (27). Some of the more common behavior change techniques included in this taxonomy are goal setting, feedback, rewards, social support, coaching, identifying barriers/problem solving, and action planning (28–31). One meta-analysis examined the effectiveness of 30 different interventions aimed at increasing physical activity to determine which characteristics are most effective (32). Overall, interventions that included established behavior change techniques were associated with greater increases in physical activity and subsequent weight loss than those that did not. Specifically, self-regulatory behavior change techniques such as goal setting, self-monitoring, and social support were associated with better outcomes (32). Some of these behavior change techniques are included in fitness technology. An analysis of seven commercially available fitness trackers (Jawbone UP24, Nike Fuelband, Polar Loop, Misfit Shine, Withings Pulse, Fitbit Zip, and Spark) revealed that most or all of these trackers included goal setting, feedback, rewards, self-monitoring, and social support (31). Fewer than half of health and fitness apps, however, include these strategies (28). Some behavior change strategies that are absent from fitness technology include those that may be most relevant to inactive populations, including action planning/implementation intentions, environmental restructuring, attitude adjustment, and barrier identification. Each of these techniques will be discussed in more detail.

### Goal Setting

Goal setting is defined by the CALO-RE taxonomy as encouraging an individual to begin or maintain changes in behavior within an intervention (27), and it is frequently included in physical activity interventions (32–35). Goal setting is included in all seven popular fitness trackers analyzed by Mercer et al. and about 40% of health and fitness apps (28, 31). When people buy a fitness tracker, they are often required to set a goal for amount of steps desired in one day. After connecting a fitness tracker to an account or smartphone application, the user will typically set a daily step goal, often with a recommendation of 10,000 steps a day. While 10,000 steps per day is the typical suggestion for healthy adults (36), there are some variations in these recommendations based on age and health status. For instance, 5,000–7,000 steps a day may be a more achievable goal for older adults or individuals with health conditions (37).

A recent randomized controlled trial used self-monitoring and goal setting to increase steps over 3 months (38). Half of the participants were asked to use a pedometer to self-monitor their walking, with goals of increasing their steps by 5% weekly. They were given an ultimate goal of reaching a 10,000-step average per day by the end of the program. The other half were provided with educational materials. The intervention group showed a significant increase in steps at 3 months and significant decreases in BMI, body fat, and waist circumference. Importantly, these results remained significant 3 months after the completion of the intervention (38). Of note, these effects may have been due to other behavior change elements in addition to goal setting. For instance, the participants were likely monitoring their walking behaviors, and social support may have played a role because participants were coworkers.

It is unclear whether steps are the best activity indicator or motivator, and whether this metric is meaningful to those without a fitness tracker or app that measures steps. Research suggests that most who own a fitness tracker are primarily concerned with monitoring their steps (39). Steps alone, however, do not provide information about exercise intensity, which may be more important than number of steps taken. For instance, people may believe they are active if they take 10,000 steps in a day, even if they are not meeting the current physical activity guidelines for level of intensity. With this in mind, a goal of a specific amount of time in different intensities of exercise (light, moderate, MVPA) may be more useful. However, steps are easy to comprehend and provide a straightforward way to conceptualize goals, while intensity is typically more difficult to gage. Distance, which is a more traditional metric, such as number of miles or kilometers walked may be another useful way to classify activity. It is important to determine whether metrics such as steps, distance, time, or frequency of exercise are differentially motivating and assess which is most likely to encourage activity increases. Future research could examine which type of goal is best for motivating individuals to be more active and to achieve recommended guidelines.

It has been suggested that small goals are more effective for long-term engagement compared to large goals. When people are successful in meeting smaller goals, they build momentum and over time are more likely to reach larger goals (40). The CALO-RE taxonomy describes small goals as graded tasks, and according to Mercer et al., none of the popular fitness trackers include these graded tasks (31). The Fitbit, however, does provide users with small goals of taking 250 steps within an hour. If the user meets this goal most hours, it becomes easier to reach the daily goal of 10,000. This may have been a new addition since it was originally analyzed by Mercer et al. These strategies require active monitoring of activity throughout the day and consistent feedback from the device. Taken together, these results suggest that goal setting is effective for increasing physical activity. While many applications and fitness trackers do include goal-setting strategies, it is necessary that individuals actively use these features, set realistic goals, and update these goals to see improvements in physical activity and health. If goals are realistic and framed properly, they can be effective in encouraging people to increase activity levels.

### Framing, Feedback, and Rewards

The content and framing of recommendations and feedback is important to consider when examining the promotion of physical activity. One recent study found that positively framed messages (e.g., “Walking can improve health”) are more effective than negatively framed messages (e.g., “Not walking enough can worsen health”) in increasing activity levels of older adults. Interestingly, young adults responded similarly, regardless of the framing of the messages (41). There may be individual or group differences in the effectiveness of different feedback messages. This information can inform how interventions are created for different age groups and may provide best practices for messages provided by fitness trackers or apps.

Feedback and rewards are other behavior change techniques closely tied to goal setting that can be effective for increasing activity (28, 34, 42–44). Feedback can be as basic as providing access to step counts, or more tailored messages designed to motivate activity. All seven trackers analyzed by Mercer et al., and many fitness apps and interventions include feedback tools such as reminders, text messages, and real-time alerts when the user has met a goal or has been sedentary for too long (28, 31). Feedback about whether or not one gets enough exercise can often encourage them to begin monitoring daily activity levels. Feedback from a fitness tracker or app can be more frequent and personalized than recommendations from a personal trainer or physician. This personalized feedback may be especially effective in encouraging individuals to monitor and change their own behaviors (45). Tracking one’s own changes in activity levels and exercise behaviors can motivate steady progress toward goals, while increasing self-efficacy.

Rewards are also often incorporated into fitness trackers, health applications, and interventions to increase physical activity when feedback shows that goals have been met (28, 46). There are a number of different types of rewards to consider. For instance, fitness trackers may vibrate, make a noise, or display a congratulatory message or friendly face when a goal has been reached. Some studies, on the other hand, have examined the implications of monetary rewards for increasing activity. One such study provided older adult participants with either monetary or non-monetary rewards for meeting their step goals across 12 5-day blocks. While participants in the monetary reward group met more step goals than the non-monetary group, both techniques were effective in encouraging participants to meet their goals, with both groups increasing their steps by an average of 108% (47).

Expanding on this line of work, another recent study examined which types of monetary rewards were most effective in increasing physical activity in overweight and obese adults. They tested a gain incentive, where participants were given money each time they met their goal, a loss incentive, where people lost money for *not* meeting their goals, and a lottery incentive, where they were placed in a lottery after meeting their goals (48). Results suggested that the loss incentive was most effective in changing behaviors, motivating more people to meet their goals than the other two conditions. Overall, rewards seem to be an effective strategy for increasing physical activity, while there may be differential effects dependent on the reward type and framing. Incorporating monetary (such as entry into a lottery or drawing if one meets their goals) or other meaningful rewards into fitness technology may lead to greater motivation and engagement in reaching individual goals.

### Social Factors

In addition to behavior change techniques that focus on the individual, it is also useful to examine the effects of social factors for increasing physical activity. Social factors such as social support or competition have been shown to increase engagement, adherence, and completion in physical activity interventions (18, 45, 49, 50). Social support could involve having a friend or family member who supports one becoming more active, or having someone with whom to walk or exercise. Those who feel supported by their family and friends are more likely to be active than those who do not (6). Further, some have found that social support for physical activity is particularly important for those who are not regular exercisers (51). This is likely why others report social support is especially effective for increasing physical activity in inactive, unmotivated adults (49). Another social factor, competition, could involve competing against another to accumulate more steps or activity. Social contact is facilitated in most fitness trackers and included in some health and fitness apps (28, 31). Such contact could include creating “teams” who encourage one another to meet their goals, or competitions in which people try to accumulate more activity than another.

About 40% of the apps evaluated by Conroy and colleagues encouraged individuals to seek support from others to help change their behaviors, while 15% of the apps facilitated comparison with another individual or group of individuals (28). All seven fitness trackers analyzed provided the opportunities for social support and comparison, allowing users to connect with others who have a similar device and create teams and/or compete with others (31). Some have suggested that both competition and cooperation are successful in increasing step counts (52). These social components, however, may only be effective if the user has friends, family, or coworkers with the same brand of device. This will allow them to connect within an app or through social media. Social support via social media has been shown effective in increasing healthy behaviors such as walking, and weight loss, and in an intervention setting can reduce attrition rates (53). People may experience fear, embarrassment, or guilt if they did not reach a goal that was shared with an audience (45). If fitness technology encouraged individuals to share their activity levels on social media, this could increase engagement and goal attainment.

Social support may be especially important for increasing physical activity in older adults (54). In individuals aged 60 years and over, having an active friend is a significant predictor of physical activity behavior (7). Others have found that social support is an important factor in exercise behaviors overall (51). Collectively, these findings suggest that social support is important for increasing physical activity and healthy behaviors, and fitness technology may be most effective when groups of people who know one another use the same device or app. Social support may be especially important for sedentary older adults or individuals who may need extra motivation to participate in physical activity. Facilitating these social connections seems beneficial for encouraging lasting changes in behavior and creating accountability for otherwise unmotivated individuals.

### Coaching

Another important strategy for increasing physical activity is coaching. This entails receiving directions or instructions from a peer or a professional. Coaching is often used in interventions and fitness technology to motivate increases in physical activity (44, 55–57). One intervention utilized professional coaching via weekly information sessions that encouraged healthy behaviors and a weekly 30-min group walking session (58). While results showed a non-significant increase in steps, there was a significant decrease in waist circumference following the 12-week intervention. It is important to note that social support may have also played a role in these positive results, as it can be difficult to separate coaching and social support. While professional coaching is often related to behavior change, it is not always feasible to provide professional coaches to large groups of people. One potential way to bring coaching strategies to larger groups of people is through peer coaching.

#### Peer Coaching

It is important to make the distinction between peer coaching and professional coaching, as there are differences between the two. Peer coaching is provided by individuals of a similar age or other demographics, or common health conditions, which can allow for more personalized support and problem-solving strategies (59). While professional coaches can provide sound advice on ways to increase physical activity, peer coaches may be even more helpful. One review found that physical activity interventions with peer coaches were just as successful as professional coaches (59). Further, the interventions with peer coaches are associated with better adherence and retention than professional coaches. This could be due to the fact that peer coaches can provide more personal feedback and relevant problem-solving strategies, which can consequently increase motivation and encouragement (59). These results suggest that fitness technology should encourage groups of peers to encourage one another to increase their activity. Perhaps those who are very active and often meet their goals could become peer coaches to those in a similar demographic and help others problem solve to meet their own goals.

#### Virtual Coaching

Another way in which coaching can be made more accessible is through virtual coaching. About 50% of top-rated health and fitness apps include virtual coaching strategies such as providing instruction and demonstrations (28). Many fitness trackers include a “mobile coach” within their connected application or website. One study examined whether virtual coaching via text messages was more effective than self-monitoring for encouraging increases in activity. In this study, 67 middle-aged adults were given a Fitbit One to monitor their own activity, and half of these participants also received text messages three times a day, which encouraged them to increase physical activity (60). The text messages led to a brief (1-week) increase in physical activity, while the Fitbit alone led to higher increases in activity at the end of the intervention (60). Since the Fitbit also provides real-time feedback and encouragement, it may be possible that both groups in this study received similar components. Participants reported that the text messages were too frequent, and therefore, they may have found them more intrusive than the Fitbit app. These frequent texts could have caused participants to become frustrated by the virtual coaching, and consequently less motivated than those with only the Fitbit. While there are many options for types of coaching, such as professional, peer, or virtual, it is important that all coaching types include sound advice that addresses barriers particular to the individual. Technology could include the option for one to become a peer coach, allowing them to give advice and motivation to individuals in a similar demographic.

### Strategies to Improve Fitness Technology for Inactive, Vulnerable Populations

Taken together, results for various behavior change strategies suggest that theoretically derived behavior change techniques are effective for improving physical activity and health. Many successful physical activity interventions include more than one behavior change technique simultaneously, and their positive results suggest that a combination of these behavior change techniques is likely most effective. It is encouraging that fitness technology often includes behavioral strategies; however, it is unclear which of these strategies people are actually using. Promoting regular and continued use of these strategies would likely lead to greater changes in activity, especially for those who are currently inactive. Behavior change interventions, fitness trackers, and smartphone apps can incorporate other strategies such as barrier identification and action planning to motivate individuals to increase activity levels. Smartphone apps are less likely to include behavior change techniques compared to trackers, especially when they are inexpensive or at no cost (28, 61). It is important for tracker or app developers to ensure that the programs that accompany these fitness trackers are derived from evidence-based techniques that elicit behavior change. While the main focus of fitness technology is often on changing behaviors, attention should also be paid to changing thoughts about one’s ability to exercise.

#### Action Planning

Many individuals who are inactive do not know how they can increase their activity. One strategy that may be especially important for this group is action planning, or prompting the user to make detailed plans about when and where they will increase their activity (28, 31). Action planning includes “if, then” plans, also known as implementation intentions (62). Such implementation intentions encourage individuals to make detailed plans about how, when, and where they will achieve their goals, which increases the likelihood that they will actually meet these goals (62). This strategy could be included in fitness technology by encouraging the user to examine their schedule for the day and determine times in which they can increase their exercise. They can then make a specific plan for how they will increase their activity. For instance, one could identify the need to go to the grocery store in the afternoon and plan to park further away from the store to get more steps. Strategies such as implementation intention interventions can help one increase both perceived control over exercise behaviors and exercise self-efficacy (63). These action planning strategies can specifically help inactive people determine the factors which prevent them from engaging in regular activity and address these issues to become more active (27). Identifying individual barriers and subsequent problem solving solutions to these barriers is rarely included in fitness trackers and smartphone apps (28, 31). With or without fitness technology, this approach could aid in changing not only physical activity behaviors but also beliefs about one’s own ability to increase their activity.

#### Environmental Supports

To motivate behavior change in sedentary populations at risk for poor health such as low-SES or older adults, environmental changes may be needed. Such changes could reach individuals who do not own, or might not be able to afford a fitness tracker. One potential avenue for these changes could be promoting or increasing community walkability. This is consistent with the 2015 “Step It Up!” call to action by the Department of Health and Human Services, along with similar programs such as WalkBoston^1^ and Walkable Communities^2^, among others. Indeed, studies have found that people who live in walkable neighborhoods get an extra 68–89 min of physical activity per week than those who live in less walkable neighborhoods (64). Further, individuals living in more walkable neighborhoods are less likely to be diabetic, overweight, or obese (65). Interestingly, these results are similar, regardless of SES. There are ways to incorporate environmental factors into technology, including providing people with maps of safe walking routes within their area. This could be done within an app that has GPS or can connect to the smartphone’s internal map. Such maps could link and incorporate census tract data to assess crime rates, safety, or air pollution within a specific area. Improving neighborhood walkability is a promising way to increase physical activity at an environmental level, with or without other technology.

In conclusion, multiple behavior change strategies may be needed to have an impact with inactive individuals. Goals should be tailored to be realistic and attainable (e.g., go for a 20-min walk once a day), with coaching (e.g., teaching people how many steps are in a mile, how much time it will take to walk 1,000 steps, or providing them with safe walking routes in their area), and social support (e.g., walking groups). For those who are inactive, additional efforts may be needed. We suggest that a focus on action planning and identifying environmental or contextual barriers, strategies that are less often included in fitness technology (63). These strategies may prove successful in groups that have difficulty increasing their activity levels, as it will encourage them to plan how, when, where, and with whom they can add physical activity.

## Accuracy of Fitness Technology for Measuring Physical Activity

In the past, many research studies on physical activity have relied on self-report instruments to assess exercise behavior (66–68). However, such subjective measures of activity can be highly unreliable, revealing both higher and lower estimates than an objective measurement (68, 69). People may overestimate activity intensity or time spent to present a favorable impression. It is also possible to underestimate activity, as many activities that are part of everyday life (running errands, walking from the parking lot to a store or office, playing with a grandchild) may not be reported as exercise, *per se*, even though steps are accumulated. Self-reports often focus on discrete activities such as going for a run or working out at a gym. Objective measurement of physical activity can record activity that may be missed in a questionnaire or self-report. This may be especially relevant for older adults who are less likely to go to a gym on a regular basis (15). Thus, there is an increased interest in using objective activity monitors. The cost, however, may be an obstacle. For example, an Actigraph GT3X—one of the most widely used instruments in physical activity research—costs about $250. Most commercially available wearable fitness trackers (e.g., Fitbit, Jawbone, Garmin) are less expensive, in the $100 range, and are more user friendly than the devices designed solely for research purposes.

Moreover, fitness trackers often provide information about activity levels and goal progress to the person wearing the device. This provides potential motivational benefits over the more expensive research models, which usually do not display information to the user. Although researchers are increasingly using consumer fitness trackers, there are some drawbacks. One major problem with fitness trackers in contrast to Actigraphy is that the algorithms that transform raw data into meaningful information are often not publicly available. Thus, it is not clear how data were configured, making it difficult to compare results across studies or to adjust based on characteristics of the participants (70). Indeed, studies have found that consumer fitness trackers have greater estimation errors for older adults with health problems or slow walking speeds and some of the different results could also be due to variations in algorithms across instruments (71, 72). There is also debate over the best locations for placement on the body, with some areas more accurate than others, and recommended placements may change with age (16, 71, 73).

### Fitness Trackers

Many studies have tested the utility of fitness trackers for measuring physical activity, with varied results. For each fitness tracker, there are many models, with a variety of algorithms that provide activity estimates. It is important to note the brand, model, and body placement used in order to facilitate comparisons across studies. Early studies found that fitness trackers including the wrist-worn Fitbit and Fitbit Ultra (74) and the waist-worn Fitbit One (75) have acceptable reliability and validity comparable to research–standard devices in the lab. The waist-worn Fitbit Zip was fairly accurate in free-living activities over a period of 7 days, though it recorded significantly more steps than the ActiGraph GT3X (76). Diaz and colleagues also found that the waist-worn Fitbit Flex and the hip-worn Fitbit One were reliable when compared to researcher-measured step counts and estimates of energy expenditure (EE) and suggest that the hip monitor was more accurate than the wrist (77).

On par with the growing fitness tracker market, researchers have begun to test and compare a variety of brands within a single study. Stackpool found that three different consumer fitness trackers (Nike Fuelband, Jawbone UP, and Fitbit Ultra) were consistent in measuring steps during treadmill walking, running, 20 min on the elliptical and agility drills. Estimates of EE, however, were less accurate (78). Others found that the step count estimates of seven commercially available fitness trackers (Fitbit One; Zip, Jawbone UP, Misfit Shine, Nike Fuelband, Striiv Smart Pedometer, and Withings Pulse) were highly correlated with the ActiGraph GT3X (79). Similarly, estimates of EE were less accurate (79). Another recent study found that the Fitbit, Movemonitor, ActivPAL, Nike + Fuelband, and Sensewear Armband Mini underestimated step counts during indoor and outdoor walking (80). In terms of the fewest percentage of errors, the waist-worn Fitbit One was comparable to the Movemonitor and ActivePAL, research-grade devices. Thus, there is growing evidence that relatively inexpensive consumer devices are generally reliable in recording daily steps, and fitness trackers seem to be most accurate when worn at the waist, especially at slower walking speeds. Nevertheless, more transparency in terms of algorithms used to calculate activity levels would be valuable for promoting comparisons across devices and studies.

### Smartphones and Mobile Applications

There has been similar interest in the reliability of smartphones as a means to track physical activity, as 68% of US adults own a smartphone (81). Hekler and colleagues tested three different Android smartphones (HTC MyTouch, Google Nexus One, and Motorola Cliq) in laboratory and free-living settings. There were strong correlations between the smartphones and Actigraphy in the lab, and moderate correlations in free-living conditions (82). A recent review summarized 26 studies that examined smartphones and apps that measure physical activity and found a large variation in the accuracy of these devices (52–100%) (83). Yet another study found promising results in the lab for step estimation with the Galaxy S4 moves app, the IPhone 5 s moves app, health mate app, and Fitbit app (84). These results show promise in the accuracy of cell phones and mobile apps in measuring steps, although clear recommendations regarding their precision and accuracy will require further investigation.

Smartphones allow for the transfer of physical activity information to applications that measure and/or encourage physical activity. There are various benefits to a smartphone application to measure and motivate physical activity, such as the fact that the user need not remember to wear or carry an extra device (85). This may be a useful intervention strategy, considering the widespread use of smartphones (81). Fong and colleagues also found that a population of older adults reported a smartphone pedometer app was more convenient than a traditional pedometer (85). However, not everyone carries their phone with them throughout the entire day, so some activity may not be recorded. While health and fitness apps are widely available on virtually all smartphones, they are rarely studied scientifically and their behavior change techniques are not always theoretically based (86–88).

Some have shown that the more expensive health and fitness applications tend to incorporate more behavior change techniques (61). Consequently, the free apps that are widely used may not offer the most effective approaches (89). While there is progress to be made in terms of available behavior change techniques within smartphone applications, many popular smartphones (Samsung Galaxy and Apple IPhone) and apps (Moves App, Health Mate App, and Fitbit App) have acceptable levels of accuracy when it comes to detecting steps, at least in the lab (82–84). Others have found favorable accuracy for smartphone accelerometers in both lab and free-living settings (90–93). Most of these studies encouraged the participant to wear the phone at the waist or hip to provide a consistent and accurate measure. Overall, smartphone apps seem to be a promising way to measure and encourage healthy behaviors (83).

Research has suggested that many consumer fitness trackers, smartphones, and smartphone applications have similar accuracy as research-grade devices for measuring multiple aspects of physical activity (steps, EE, etc.). Beyond personal use, fitness trackers hold promise as more than a measurement tool in research studies. They can provide valuable tools in interventions that promote behavior change. It is important to consider the population being measured, as devices may vary in utility depending on personal characteristics. For instance, the Fitbit had estimation errors above 60% within a population of older adults with reduced mobility (71). It remains important to consider and report device placement when comparing step counts or energy estimates between studies and individuals (16, 71, 77, 80).

With these caveats in mind, many researchers continue to use Actigraphy to measure activity. While the Actigraph is well validated, it does not provide information to the user about their activity levels. However, an Actigraph does allow the researcher more control over what information is provided to the person wearing the device. A fitness tracker or smartphone application allows the wearer to view their daily step counts, distance walked, calories burned, among other metrics. This can be reinforcing and help them determine whether or not they have improved or met a certain goal. Thus, intervention studies can use this technology to encourage participants to self-assess their changes in daily activity. This opens up possibilities for behavior change involving cognitive-behavioral components such as goal setting, real-time feedback and rewards, social support, and coaching.

## Behavior Change Interventions with Technology

Beyond measuring physical activity, if used correctly and for long enough, fitness trackers have the potential to facilitate long-term behavior change. Although fitness trackers are similar to pedometers, there is evidence that fitness trackers are more effective in increasing activity (94). A pedometer is a device that strictly measures number of steps taken per day, while a fitness tracker utilizes a more advanced accelerometer that can provide estimates of not only steps but also EE, distance walked, elevation, heart rate, diet, sleep quantity, and quality, among other things. Fitness trackers also have the capability to connect to computer and smartphone applications, allowing the user to view their progress and goal attainment over time. Access to this daily data encourages users to keep track of their exercise behaviors throughout the day and over longer periods of time. Such self-monitoring is associated with increases in activity and weight loss (33, 34, 95). One recent intervention examined changes in activity levels using either a pedometer or a Fitbit paired with a website to self-monitor their activity. Results suggested that not only did those who wore the Fitbit exhibit greater increases in activity, they were more satisfied with the device (94). This could have been a result of the various behavior change techniques included in the Fitbit and companion website, or the data that a fitness tracker provides above and beyond step count.

Another recent randomized controlled crossover trial sought to address whether a fitness tracker is more effective when combined with a physical activity program developed by NIA/NIH: Go4Life (96). The intervention group was provided with a Fitbit, from which they received feedback, along with Go4Life educational material/counseling for 24 weeks. The control group was given the Fitbit with no device feedback for 24 weeks. The groups then switched for another 24 weeks. Overall, while both groups lost weight, there were no significant differences in activity levels, weight, or body fat between the groups (96). These results suggest that the Fitbit alone, with no feedback, had similar results as when it was combined with the Go4Life counseling program. Results should be interpreted with caution when generalizing to consumers outside of a controlled experiment. The control group received no feedback from the Fitbit, which is not the case when using it in typical daily life. It is also possible that wearing the Fitbit (even with no feedback) led to increases in physical activity because participants knew they were being monitored. As noted above, an important issue when using a fitness tracker as part of a research study is that having a fitness tracker could actually be an intervention in and of itself.

Jakicic and colleagues recently examined the effectiveness of a lifestyle intervention with or without a fitness tracker. Two groups received recommendations for dietary restrictions, physical activity increases, and group counseling (97). Six months into the intervention, half of the participants were provided with a fitness tracker (SenseWear Pro Armband) and the online support paired with the device. The other half were able to self-monitor their activity and diet on a study website. Interestingly, the group that wore the tracker lost less weight than the group who did not. Changes in physical activity between the two groups, however, were not significantly different. These results suggest that fitness trackers may not be more beneficial than a theory-based physical activity intervention. Authors suggest that the consistent reminder of goals from the fitness tracker may have discouraged those who were unable to meet these goals, and therefore decreased motivation (97). Because the device was worn on the upper arm, these results may not be generalizable to a wrist-worn device. It is possible that a wrist-worn device could give the user information about their activity that they can easily see throughout the day. This could cause consistent self-monitoring and may remind individuals throughout the day to increase their activity. This information may not be as easily accessed on the upper arm. It is also possible that because the intervention was very similar to the control group other than the addition of the tracker, both groups received similar behavioral support.

Other studies have examined changes in activity with fitness tracking in a more natural situation. One such study by Rowe-Roberts and colleagues invited full-time office workers to purchase a Fitbit Ultra at a 20% discount. About 40% of the 556 employees invited purchased the Fitbit (98). These 212 people increased their daily physical activity and decreased their diabetes risk after 7 months, even without any additional intervention. This situation is similar to what would be encountered in an everyday setting, as participants purchased the device themselves and were allowed to view their daily step counts (98). Participants were also able to utilize behavior change techniques included in the Fitbit app and website, such as goal setting and social networking. Such social networking tools included the ability to view other employees’ step counts, create teams, and challenge each other to take more steps. Approximately 80% of participants worked at the same location, so many likely used these social tools. This may have played a crucial role in the success and continued use of the fitness trackers in this study.

It is possible that the monetary investment in the device may facilitate changes in behavior and subsequent health risk. There may be motivational differences between one making a conscious decision to go out and buy a fitness tracker versus signing up for a study and receiving one. The decision to buy a fitness tracker implies some degree of motivation to make healthy changes in their behavior. It could also be possible that making a financial investment in a fitness tracker could lead to a greater motivation to actually change behaviors. Such factors introduce many challenges in terms of a research study and suggest the need to investigate whether these differences exist, and which aspects of these devices and what motivational factors are most successful in facilitating behavior change.

While it seems that using a fitness tracker alone may be sufficient in facilitating behavior change, there are other factors to consider (96). Some interventions which incorporate extra motivational techniques are more effective than fitness technology alone, suggesting that other strategies may be needed to facilitate lasting behavior change. There are extra challenges that arise when using fitness technology daily. Some of these challenges include remembering to wear the device daily and assessing battery life (17). Depending on the device, the battery may need to charge weekly, or the battery may need to be changed every few months. More importantly, users need to be able to consistently sync, view, and understand the information provided by the device (17). These trackers are likely most effective when the person wearing the device actively uses the smartphone app or website that includes theoretically driven behavior change techniques. This highlights another research question; whether people are actively using the applications that are associated with a fitness tracker.

## Factors Involved in Behavior Change

Overall, it is still not well understood whether the behavior change techniques included in fitness technology are sufficient for changing behaviors over the long term. Research is needed to determine which motivational components are most effective for long-term increases in activity, and what combinations of these components work best for increasing physical activity. Behavior change strategies should be tailored to specific groups such as older adults or low-SES individuals who are inactive or believe they do not have time to exercise. If having fitness technology is not enough, it becomes important to determine which other components can influence successful behavior change.

### Duration of Effects

Few intervention studies using fitness technology have reported follow-up data, and consequently little is known about the duration of the changes in activity (99). Future research should aim to collect data that evaluate the programs or features associated with long-lasting increases in physical activity. Such research could also examine factors that influence dropping out from studies. This knowledge could lead to improvements in fitness trackers and smartphone apps and increase the likelihood that this technology will make a lasting impact on nationwide health. It may be difficult to measure duration of effects if individuals stop using fitness technology with time. If the goal of the user is to monitor steps taken in a day, the device itself may become less useful over time as they learn how many steps they take in a typical day or on a particular route.

When monitoring usage with apps, it may look as though someone is inactive, when in fact they are taking their usual walking routes without wearing the device or carrying their smart phone. This issue should be investigated before concluding that people give up on their walking routines based only on monitoring software. While using fitness tracking technology can lead to increases in self-monitoring, this may lose relevance over time when people get similar amounts of activity each day (100). Fitness trackers have the potential, however, to increase feelings of control over one’s own exercise behaviors (100). This could actually increase the likelihood of sustained behavior change, as feeling in control of exercise behaviors can increase feelings of self-efficacy – a key predictor of physical activity adherence (24). Consideration of these factors is important in both the development and refinement of fitness tracking technology. It is possible that someone will stop using this technology when they have already developed healthy habits and no longer need the device. Research should aim to discover ways to increase control beliefs and self-efficacy for exercise with fitness technology, which will likely facilitate long-term increases in physical activity.

### Obstacles to Increasing Physical Activity

Recent reports have suggested that over 20% of Americans own a wearable fitness tracker (101, 102). However, it is estimated that only half of these individuals wear the device daily (101). The demographics of those who own a fitness tracker seems to be fairly narrow. While a 2014 report suggested people aged 30 years or below are 55% more likely to own a fitness tracker than those who are over 30 years (101), more recent reports suggest that adults aged 35 years and older are quickly adopting this technology (103). Those who do own a fitness tracker tend to be more affluent, educated, and familiar with technology than individuals who do not own one (101). Others have found that males, inactive, and unemployed individuals are less likely to own a fitness tracker (39). These narrow demographics are somewhat worrisome, as those who are older and less educated are at higher risk for a sedentary lifestyle and health problems than younger, more well-educated adults. It is encouraging, however, that data suggest that interventions with fitness trackers are most effective in increasing daily physical activity of people who are inactive compared to those who are already active (38, 104). This highlights the importance of bringing fitness technology to these vulnerable populations.

It remains important to determine how to identify and motivate individuals who are at the greatest risk for physical inactivity or poor health. Those who are sedentary and not actively considering increasing their activity are difficult to recruit for exercise studies and may not be interested in using fitness technology. Further, these individuals may believe that such technology would not help them increase their activity, or have no motivation to increase their activity (39). This highlights the need to develop interventions or technology that addresses the needs of these individuals who are not motivated to develop healthy behaviors and have little desire to make lifestyle changes. This lack of motivation could be a result of low self-efficacy for exercise behaviors, which is often closely related to physical activity levels (25, 105). Recently, it has been suggested that incorporating techniques such as motivational interviewing may increase motivation and self-efficacy (106). Motivational interviewing is a form of counseling aimed at prompting increases in self-efficacy, which may leave people more open to and invested in changing their behaviors (107). Motivational interviewing techniques, however, are not currently included in fitness technology (28, 31). Fitness technology could potentially utilize motivational interviewing techniques when a user is creating an account. Interventions or technology geared toward unmotivated individuals should focus on planning and implementing ways to help them change their behaviors, including identifying barriers, action planning, and modifying environmental factors (106).

#### Demographic Factors

There are specific barriers and obstacles that prevent some groups from getting enough exercise. Demographic factors such as gender, age, SES, or education are associated with amount of physical activity engagement (6). As such, young adults are more active than older adults, younger men are more likely to be active than younger women, and older women are more active than older men. Further, people with lower SES based on level of education or income are more likely to be physically inactive (6, 108). These variables should be taken into consideration when designing interventions or improving fitness technology. If individuals with these characteristics are particularly vulnerable to being inactive, research should determine ways to make fitness tracking technology affordable and accessible to these populations. Researchers and app developers should consider how this technology can address the unique barriers these groups face.

A smartphone app may be an ideal way to reach large numbers of people, as about 68% of American adults currently own a smartphone (81). However, those who are older and of lower SES are less likely to own smartphones. Approximately 30% of adults aged 65 years and older own a smartphone, while 86% of adults aged 18–29 years own one. When ownership is broken down by education, 81% of adults who have graduated college own a smartphone, while only 41% of adults who did not graduate high school own one. With regard to income, 52% of individuals who make less than 30,000 per year own a smartphone, and 87% who make more than 75,000 per year own one (81). While these important gaps exist, the percentage of low-SES and older adults who use smartphones is growing (81). Perhaps in the near future, efforts to increase availability and theoretical basis of smartphone applications will be an ideal way to bring technology to these inactive groups.

#### Psychosocial Factors

Psychological barriers such as low motivation for exercise or self-consciousness may prevent inactive individuals from regular exercise (6). Further, perceived lack of time is consistently cited as a barrier to physical activity. Inactive individuals who have little time may not know when or how they can incorporate physical activity into their day. Technology that address these key issues may be especially important for encouraging behavior change in busy, inactive adults. Others have posited that sense of control and self-efficacy are influential predictors of physical activity (108). It has also been suggested that social factors play a role, as social support, strain, and social networks can influence health behaviors and outcomes (108–110). Further, these social factors may not be useful for those without friends or family members with the same device or app. While many trackers and apps do include social factors (31), few specifically target changes in self-efficacy and control or other attitudes and beliefs.

With these factors in mind, it may be most beneficial to encourage large groups of people to become more active, and their increased activity could affect others in their social networks. A focus on increasing self-efficacy and control beliefs may be integral in changing behaviors of those who do not believe they are able to increase their activity. In an activity intervention program with sedentary older adults, Jette et al. used cognitive restructuring techniques to increase self-efficacy and control over physical activity in conjunction with an in-home resistance training program (111). It is also possible that although changes in self-efficacy are not specifically targeted, if the user sees that they can consistently meet their goals, self-efficacy could increase. Acknowledging and addressing the barriers that particularly affect inactive groups will make technology useful to more people. Other techniques such as providing specific information about when and where to exercise may be especially valuable for those who have led a sedentary lifestyle for many years. Unfortunately, such strategies are not prevalent in many popular fitness trackers (31).

Common barriers for low-SES individuals include a perceived lack of ability, social and physical discomfort, lack of motivation, shortness of breath, as well as environmental obstacles (112). Programs or fitness trackers could include problem-solving techniques to determine individual barriers to initiating physical activity and help these inactive adults overcome their unique barriers. Similar barriers for older adults include fear of falling, poor health, perceived lack of time, or negative affect such as depression or embarrassment (7, 113–115). A low-impact exercise such as walking has been shown to reduce fear of falling, and even decrease actual incidence of falling, one of the most cited concerns among inactive older adults (113, 115–117). Another potential psychological barrier for older adults could be an apprehensiveness toward technology. Research has shown that although older adults may be initially wary of fitness technology, after consistent use they report they find the technology useful and acceptable (46). This age group may, however, need help setting up the device and learning how to interpret the data. Given that many older adults have low expectations about their ability to exercise (118), strategies that increase self-efficacy for exercise may be especially beneficial for this group.

#### Environmental Barriers

There are environmental barriers that can interfere with physical activity behavior that should be addressed with or without fitness technology. These barriers include inclement weather, lack of facilities, unpleasant scenery, unfavorable terrain, perceived neighborhood safety, or physical activity behaviors of others in their neighborhood (6). These barriers are especially relevant for vulnerable groups, such as older adults and individuals of lower SES. Low-SES individuals are less likely to live near a gym or facility to engage in physical activity (119). Even when they are close by, these facilities typically have a monthly fee that can be expensive. Walking may be an ideal strategy to increase physical activity in this population, given it is a free activity. In fact, one study found that walking is the preferred type of exercise for those with lower SES (112). Low-SES neighborhoods, however, may be associated with safety concerns such as higher crime, unleashed dogs, poor snow and ice removal, broken sidewalks, and unpleasantness of scenery (120). Wilson and colleagues discuss that while data do not necessarily support the validity of these perceptions in the area of Canada they surveyed, these factors remained commonly cited as barriers to engaging in physical activity in low-income neighborhoods. Further evaluation of these neighborhoods revealed that low-SES neighborhoods also have little or no access to walking paths (120). Addressing these unique barriers including environmental factors may be key in motivating low-SES populations to increase their physical activity. Perhaps fitness technology could prompt the user to identify what holds them back from engaging in regular activity, with factors like time, environment, and motivation as options. This allows for the tailoring of specific recommendations that will be useful for people who need help changing their behaviors. Individuals could also be directed toward walking trails or parks within the area.

## Summary, Conclusion, and Future Directions

The importance of regular physical activity is well documented and well publicized (1, 121, 122). There is a disconnect, however, as only a small percentage of adults engage in the recommended amount (4). This is particularly so for older adults and those from low-SES backgrounds (4). A key question is how to change behaviors to increase and sustain physical activity, especially for those who are sedentary. The use of technology, including fitness trackers and smartphone apps, show a great deal of promise for measuring and encouraging physical activity. In addition, when these devices are combined with behavioral strategies, they show greater benefits (28, 31, 61). While research has begun to answer some of the important questions about how fitness technology can increase physical activity, important gaps in the literature remain.

Research supports the usefulness of activity trackers for measuring steps, while other metrics such as EE may not be as accurate (78, 79, 123). There are still some inconsistencies in estimates of physical activity and sleep over 24 h, suggesting that improvements are still needed (124). The best placement for wearing these devices, i.e., whether monitors are more accurate when worn on the wrist or the waist is also not resolved. While research tends to favor the accuracy of the hip placement (77, 80), consumers seem to prefer the wrist-worn monitors (39). There is also some uncertainty remaining with regard to accuracy as walking speed decreases (22). These estimation errors may be most important when considering activity in older adults. As the algorithms used by these devices are not publicly available, it is unclear whether they take age into account as more sophisticated research devices do. Future app and fitness technology developers should take care in ensuring the accuracy of their algorithms with different populations. Other unanswered questions include whether steps are the most useful metric for measuring and motivating activity. More research is needed to examine ways to motivate inactive individuals to be more active, to determine whether fitness technology leads to lasting changes in behavior, and why people stop using this technology. We examine the implications of these questions and provide some suggestions for how these questions can be answered with future research.

### Increasing Physical Activity in Sedentary Populations

For personal use, it is still unclear whether using a fitness tracker alone leads to long-term behavior change, or if it is dependent on other sources of motivation. While many trackers and health and fitness applications incorporate established behavior change techniques, it is important for research to determine which aspects of these devices are most effective and which are being used. A device could include behavior change techniques that work in an intervention, but utilizing these resources daily is likely needed to have an effect in fitness technology. While many fitness trackers and health applications do incorporate evidence-based behavior change techniques, most have room for improvement. Those strategies (e.g., barrier identification, action planning, environmental restructuring) that are likely to be most useful to inactive or unmotivated individuals are absent from many fitness trackers and smartphone applications (28, 61). While apps that cost money often have one or more behavior change techniques, many free smartphone apps lack strategies like goal setting, self-monitoring, rewards, social support, and coaching (28, 61, 88).

There is a need to identify the key barriers that are especially relevant for older adults and those of low SES and to provide suggestions to overcome these barriers within fitness technology (61, 125). While fitness trackers typically include multiple established behavior change strategies such as goal setting, self-monitoring, social support, and rewards, other missing strategies may be particularly relevant for sedentary adults (27). Identifying barriers that prevent individuals from regular physical activity, and helping them to plan where, when, and with whom they can increase their activity may be especially important (31). These features are among the biggest opportunities for improvement in fitness technology and will increase the usefulness and relevance for inactive populations.

Many have suggested that app developers, health researchers, and behavior change experts should continue to collaborate to develop technology that includes multiple evidence-based strategies that encourage physical activity (28, 31, 61). Such a multidisciplinary approach will allow for development of apps with multiple behavior change techniques that will likely have the greatest potential of changing behaviors over the long term. Behavior change experts could develop guidelines that help app developers understand which techniques will be most helpful for their specific app or intended population (61). The CALO-RE taxonomy would likely be useful for such an effort (27). App developers could also help behavior change experts develop apps with a user-friendly design that is easy to navigate, with clear data and recommendations. Such an app should be created by listening to feedback from the intended population. It is important to continue testing the effectiveness of fitness technology for changing behavior, which will help determine the best practices for both development and improvement. Such research and collaboration will ensure that the millions of apps and fitness technology used daily provide sound recommendations and strategies for increasing physical activity.

### Making Fitness Technology Accessible

Encouraging inactive populations to increase their physical activity is an important public health consideration. If fitness tracking technology has the potential to encourage long-term increases in physical activity, making it readily available is an important goal. This is especially true for those who do not have financial resources to buy a tracker, or do not own a smartphone that is capable of downloading a fitness app. There are some viable options for providing fitness trackers to large, diverse groups of people throughout the country. For instance, many companies have started providing fitness trackers to their employees for free or at a reduced price. The social components available in these devices have the potential to foster a greater sense of teamwork within the workplace. This may be especially relevant for low-SES individuals or older adults who are in the workforce. In one study, fitness trackers increased productivity and decreased the amount of sick days taken (126), suggesting the powerful potential if adopted by more companies. Fitness trackers have the potential to improve the well-being of employees, while also being useful in increasing productivity and workplace happiness, which is beneficial for both employees and employers.

Another possible solution for making fitness trackers more readily available could be in healthcare settings. Recent reports suggest that many doctors are now prescribing exercise to their patients with chronic health conditions such as diabetes, hypertension, or cardiovascular disease (127). Fitness trackers can be a successful component in a healthcare intervention, increasing physical activity while decreasing blood pressure and BMI (128). If fitness trackers were covered via health insurance, it could help both doctors and patients track day-to-day exercise, along with other metrics like diet, heart rate, or sleep. Such programs may be able to connect doctors to patients in ways that were not previously possible (77). This type of daily monitoring may also provide the potential to personalize healthcare tracking and recommendations (129). This may be particularly helpful for an older adult population, for whom physicians seem to play a key role in the initiation and maintenance of healthy behaviors such as low fat diet and physical activity (8). Low-SES individuals seem to place a similar weight on physician recommendations, as a prescription for exercise from a physician is often cited as a facilitator of physical activity (112). While it may seem to be a costly investment to provide these monitors to large numbers of people, it may be an investment that saves billions of dollars in healthcare costs each year. The CDC estimates that physical inactivity is associated with approximately $117 billion dollars per year in healthcare costs (130). Health insurance companies could potentially fund these devices as a form of preventative medicine, much like they often provide a stipend for a gym membership.

### Using Fitness Technology in Intervention Research

Fitness technology has the potential to revolutionize physical activity research, allowing large-scale interventions to be conducted that gather real-time data about activity levels, sleep, and nutrition across the globe (76). An important consideration in using a fitness tracker in a physical activity intervention is the fact that it is currently unclear whether giving someone a device is an intervention in itself. This could be problematic in an intervention study in which both an intervention and control group both wear a fitness tracker. The control group is likely still using the strategies included in the device such as goal setting and feedback, which makes it difficult to determine whether or not this is a true control group. Further, such a control group may increase their activity more than if they were not wearing the tracker, potentially because they know they are being monitored. Indeed, it could be that giving someone a fitness tracker is in itself a behavioral intervention. It is also possible that there could be a self-selection bias for individuals who use or sign up for a study that uses a fitness tracker, the individuals who want to be in a physical activity intervention may be more active and motivated than the general population. If the goal is to recruit inactive individuals, there may need to be strict exclusion criteria that take baseline activity levels into account.

One potential way to utilize technology in an environmental intervention comes from the MapMyFitness App^3^. This app has made data public, which includes information about users, workouts, and routes that allow for analysis of change in exercise behaviors over time and geographic locations. With over 20 million users and 70 million routes, it can highlight areas with superior walkability, and areas where little physical activity takes place. In terms of intervention research, these data could be used to establish baseline activity levels within a specific location or examine common workouts within a particular area (131). Further, information about users within a specific location such as age, gender, and BMI can be assessed. Such technology allows researchers to determine which areas are most active or inactive and examine changes that occur after environmental interventions have taken place.

Fitness technology may also be financially economical for research because of the reasonable cost. Further, this technology may provide an invaluable intervention tool, because they provide information about goals and self-monitoring of progress, while research-grade monitors such as the Actigraph do not (79). Self-monitoring of progress and goal achievement may lead to increases in self-efficacy and sense of control for exercise, which could encourage long-term lifestyle changes (132). Fitness devices also make it possible to create and set goal reminders and facilitate social interventions such as creating competitions and/or teams. Fitness technology can simultaneously measure many health factors at a low cost, while allowing a participant to monitor changes in their own behavior.

Some of the drawbacks and limitations of these devices include the absence of publicly available algorithms, and reproducibility issues as devices and software are continually evolving. If measuring physical activity with smartphone apps, the brand of device should be taken into account as there may be measurement differences between different types of smartphones. It must be made clear whether or not using such a device is an intervention within itself, or if it is most effective when combined with other components. Even with these caveats in mind, fitness tracking technology seems especially beneficial for physical activity research.

### Final Conclusion

Overall, many behavior change strategies are included in fitness technology. However, it is not clear how often these features are regularly used. There is the potential for sustained increases in physical activity; however, many strategies are missing that would be most helpful to individuals who are currently inactive. More work needs to be done to determine under which conditions using fitness technology can facilitate behavior change over the long term, and developers should use this knowledge to improve the technology.

Future work should ensure that fitness technology continues to include theoretically derived behavior change techniques that are useful for their intended population. Strategies such as goal setting, self-monitoring, feedback, rewards, social support, and coaching seem to be especially helpful in increasing activity and healthy behaviors. We recommend adding other strategies such as identifying obstacles, restructuring negative attitudes, action planning, and modifying environmental factors to motivate inactive populations who may not know how, when, where, and with whom to start increasing their activity levels. Regardless of the type of intervention, an effort should be made to address the particular barriers that keep inactive people, especially older adults and low-SES populations, from engaging in regular physical activity. While there are many questions that remain answered, the public health implications of using fitness technology to promote behavior change is quite promising.

## Author Contributions

This is a collaborative research in which both authors contributed equally to almost all parts of the review; AS and ML contributed equally to the conception; AS performed the literature search; and AS and ML wrote the manuscript.

## Conflict of Interest Statement

The authors declare that the research was conducted in the absence of any commercial or financial relationships that could be construed as a potential conflict of interest.

## Footnotes

^1^http://www.walkboston.org/.

^2^http://www.walkable.org/.

^3^http://www.mapmyfitness.com/.
